# Latent Classes of First Incident Difficulty in Instrumental Activities of Daily Living

**DOI:** 10.1093/geroni/igab046.3707

**Published:** 2021-12-17

**Authors:** Danielle Feger, Jennifer Deal, Alden Gross

**Affiliations:** 1 Johns Hopkins University, Baltimore, Maryland, United States; 2 Johns Hopkins Bloomberg School of Public Health, Baltimore, Maryland, United States

## Abstract

Ability to perform instrumental activities of daily living (IADLs) deteriorates during prodromal Alzheimer’s disease (AD), eventually leading to impaired everyday functioning and dementia. Ordering and timing of IADL difficulty onset may identify individuals at greater risk of cognitive impairment, but most studies only consider total number of difficult tasks. Leveraging longitudinal data from the Advanced Cognitive Training in Independent and Vital Elderly (ACTIVE) Study who entered free of any IADL difficulty (N=1266), we hypothesized that a latent class analysis based on timing of first reported IADL task difficulty would reveal class differences in cognitive functioning . Participants were followed until they self-reported at least one IADL difficulty, study completion (10 years), or loss to follow-up. Discrete-time multiple event process survival mixture (MEPSUM) models were used to simultaneously estimate hazards of incident IADL task difficulty across 7 task groups. Two, 3, 4, and 5 latent class models were fit to the data. Both unadjusted and covariate-adjusted models (adjusted for age, sex, race, education, marital status, and general health rating) were fit. Using the 2-class solution as the most parsimonious model, model entropy was 0.855. The model was able to distinguish a class of participants with lower global cognitive factor scores at baseline (Cohen’s D = 0.23, P = 0.04). We conclude that first incident IADL difficulty may be a useful measure in identifying individuals with worse cognitive functioning.

